# The statistical significance revolution

**DOI:** 10.1093/jncics/pkae035

**Published:** 2024-04-29

**Authors:** Alfred I Neugut, Tito Fojo

**Affiliations:** Department of Medicine and Herbert Irving Comprehensive Cancer Center, Vagelos College of Physicians and Surgeons, Columbia University, New York, NY, USA; Department of Epidemiology, Mailman School of Public Health, Columbia University, New York, NY, USA; Department of Medicine and Herbert Irving Comprehensive Cancer Center, Vagelos College of Physicians and Surgeons, Columbia University, New York, NY, USA; James J. Peters Department of Veterans Affairs Medical Center, Bronx, NY, USA

## Abstract

Statistical significance has long relied on the criterion of *P* less than or equal to .05. Although this threshold has generally functioned well, it has engendered some negative practices to circumvent it and been criticized as too inflexible. We concur with the statisticians and methodologists who are currently arguing for more flexibility to the *P* value and more reliance on the 95% confidence interval, a shift that is likely to change future practice in data analysis and interpretation for oncology.

A key part of any experiment or study is the comparison of two interventions: How do treatments A and B compare with respect to an outcome, such as overall survival? For such a question, we conduct experiments with the initial presumption that there is no difference between the two entities, referred to as the *null hypothesis.* We perform statistical tests to estimate the probability that the observed difference may have occurred by chance and often choose a probability value, or *P* value, of .05 as the acceptable level of uncertainty, a concept most physicians grasp.

How was this level selected? In 1925, Ronald Fisher, a British statistician, selected .05 as a reasonable level at which to reject the null hypothesis. Fisher described his initial investigations in one of his books. In the early 1920s, while a statistician at an agricultural research station near London, he met Blanche Bristol and her fiancé William Roach, two botanists. Sitting down for tea one afternoon, Mr Fisher poured tea for the lady, adding milk first. Dr Bristol demurred, stating that she preferred pouring the tea first. Believing the order of pouring should make no difference, Mr Fisher was skeptical, but Dr Roach said, “Let us test her.” On the spot, Mr Fisher designed an experiment, giving Blanche Bristol 8 cups—4 with milk poured first and 4 with tea poured first, sorted randomly. As there were 70 possible combinations of 4 out of 8, with none more than once, the probability that Blanche Bristol would guess all 4 cups correctly was 1 in 70 (0.014). For the analysis, Mr Fisher devised the Fisher exact test, which is still used today. The small audience that gathered watched Dr Bristol correctly select the cups. How she discerned them remains unexplained, despite many articles written about this episode, but we can safely conclude that the British take their tea very seriously!

Impressed with Dr Bristol’s success, Mr Fisher continued to mull the tea experiment and realized that the likelihood she could have guessed 3 cups correctly was 16 of 70 (23%), a level of accuracy that would have made him less confident in her ability. He also realized that his confidence would have grown had he used more cups. This episode enabled him to clarify the components of a good experiment—sample size, control groups, randomization, and statistical analysis—previously not routinely included in experimental designs.

Mr Fisher’s 1925 book *Statistical Methods for Research Workers,* has been influential in statistics and biomedical research. In it, he introduced the concepts of the null hypothesis and statistical significance, including use of .05 as the threshold, a value he apparently chose from the bell curve and the area of the 2 tails when applying 1.96 SDs from the mean (1,2). And 100 years after the tea-break experiment, .05 has acquired widespread acceptance as the arbiter of statistical significance, becoming the binary decisor of whether a difference is “real.” Its applications are widespread, and although the value dominates studies, reports, journal decisions, and grant applications, support for its use and value is far from universal (3-6).

Two hypotheses, the null and the alternative, whose testing is fundamental to statistics, provide a “method” for reaching decisions based on the data analyzed. In epidemiology, for example, the null hypothesis represents no association between the factors or characteristics being investigated. With sex and a common disease as one example of factors or characteristics under investigation, the finding of “equal prevalence amongst males and females of the given disease” leads to the conclusion that no association exists between sex and the disease. Similarly, in oncology, the experimental data are often benchmarked against a null whose veracity is not under question and that represents no effect of the intervention on the treatment outcome. Importantly, in these cases the *P* value does not describe the likelihood that the null hypothesis is true or false. Rather, it provides information as to the probability that the data observed (or more extreme data) would be seen *if the null hypothesis were true*. Those who argue against thresholds such as *P* less than or equal to .05 favor seeing *P* values as a continuum, with the likelihood the results occurred by random chance less likely the smaller the *P* value is, that in turn provide stronger evidence for rejecting the null hypothesis.

Furthermore, *P* values have nothing to do with the magnitude of a therapy’s benefit, only its reproducibility—be it a small or large difference. Its numerical value, below .05, is arguably largely irrelevant. Speakers often point proudly to *P* values with many zeros that were achieved with large numbers of patients but small effect sizes, believing that their results are better than those in smaller studies with truly meaningful benefit but *P* values of “only” .05. Unfortunately, those many zeros only tell us that the small benefit observed is more likely just that: a small benefit.

Recently, both supporters and opponents have questioned the reliance on *P* less than or equal to .05 as an absolute or universal criterion for scientific validity (3-6). Let us highlight some problems mentioned with regard to statistical significance by beginning with an example. Consider a trial with median survivals of the experimental and placebo groups of 13.8 and 10.9 months, respectively, and *P* less than or equal to .08, judging the intervention not more effective. Would it get published? Likely it would, albeit in a less prestigious journal. The authors might argue for a “trend” to the experimental treatment having more efficacy but not conclude that it “worked.” Would you consider the experimental therapy an option for a loved one? Part of the problem arises from overlap and ambiguity between the concepts of effect size or true significance and statistical significance. When Fisher adopted .05, it does not seem that he intended it to become an inflexible, all-or-none significance barrier. Thus, we should avoid concluding that there is no association or difference simply because the data did not achieve *P* less than or equal to .05.

This point is critical in terms of how our thinking has to evolve. We need to rely on the 95% confidence interval rather than the *P* value because it enables us to assess how our risk estimate approaches the 2 ends of the 95% continuum. We can weigh this value along with other evidence from the literature to determine whether a risk estimate that misses statistical significance by a small amount is still a meaningful finding, especially if the risk estimate is robust.

Additionally, increasing pressure on those conducting clinical trials has led to questionable efforts to find *P* less than or equal to .05 or imply significance where there likely is none. Examples include 1) analyses of subcohorts, 2) serial analyses stopping when *P* less than or equal to .05 is achieved, 3) continuing data collection until results achieve statistical significance, 4) implying that statistically nonsignificant results describe a “trend” (6), and 5) delaying decisions on including outliers until initial analyses have been completed and selectively excluding study patients for dubious reasons. The problem of multiple testing, including the problem of multiple testing *relying on or driven by* the goal of achieving a “significant *P* value,” has emerged as especially problematic in the context of analyses that look for correlations with genomic findings.

Of course, statistical significance has also served us well in many respects. Begg (7) has noted good reasons why *P* less than or equal to .05 has been widely adopted and withstood the test of time. Fundamentally, *P* less than or equal to .05 is a simple test, universally understood, that provides clinicians without statistical training thresholds that studies must achieve and a reference point for judging findings. Exchanging this long-accepted, universally understood criterion for a more flexible, possibly more arbitrary and subjective judgment of positive vs negative, cannot be undertaken lightly.

We endorse the recommendations of Amrhein and colleagues (8), signed by 800 statisticians and epidemiologists, calling for abandoning the term *statistical significance* in describing study results, more sparing use of *P* less than or equal to .05 as the criterion for decision making, general reliance on 95% confidence intervals, and more thoughtful consideration of study results.

A gradual decline in reliance on *P* less than or equal to .05 has occurred in epidemiology and psychology, and it is time oncologists embrace it by shifting our emphasis from *P* values to magnitude of benefit and moving beyond .05 as the inflexible decisor of success, instead embracing a willingness to accept higher *P* values. This shift will require changes in the design and conduct of studies and some overhaul of the drug-approval process. We will need to begin by agreeing on what constitutes meaningful magnitudes of benefit, appropriate for the disease and the clinical setting and finding guidance in a decade-old American Society of Clinical Oncology publication that was never pursued and in the European Society for Medical Oncology Magnitude of Clinical Benefit Scale (9,10). Meaningful magnitudes of benefit will require fewer patients, allowing for more rapid completion of trials, and will not be judged by prespecified *P* values. This change will benefit both the development of therapies for more common cancers but especially help the conduct of trials with targeted therapies for tumors with specific molecular profiles. Wide deployment of molecular analyses has redefined and increased the number of cancers we now define as “rare” but that often achieve meaningful magnitudes of clinical benefit—such as drugs targeting EGFR, ALK, MET, RAF, and NTRK. Some may argue that moving beyond .05 as the inflexible decisor of success could lead to the approval of less worthy therapies, but we would counter that this would be unlikely with a paradigm that involves both the magnitude of benefit and a *P* value, albeit a somewhat flexible one. In this way, we will ensure approval of therapies with truly meaningful magnitudes of benefit, reducing trial size; allowing for the conduct of more trials, all of which will be completed more quickly; and reducing drug development costs and spiraling drug prices.

## Data Availability

No new data were used or generated for the writing of this commentary.
